# Synthesis of magnetic nanofibers using femtosecond laser material processing in air

**DOI:** 10.1186/1556-276X-6-375

**Published:** 2011-05-06

**Authors:** Mohammed-Amin Alubaidy, Krishnan Venkatakrishnan, Bo Tan

**Affiliations:** 1 Department of Mechanical Engineering, Ryerson University, 350 Victoria Street, Toronto, ON, M3N 2H8, Canada; 2 Department of Aerospace Engineering, Ryerson University, 350 Victoria Street, Toronto, ON, M3N 2H8, Canada

## Abstract

In this study, we report formation of weblike fibrous nanostructure and nanoparticles of magnetic neodymium-iron-boron (NdFeB) via femtosecond laser radiation at MHz pulse repetition frequency in air at atmospheric pressure. Scanning electron microscopy (SEM) analysis revealed that the nanostructure is formed due to aggregation of polycrystalline nanoparticles of the respective constituent materials. The nanofibers diameter varies between 30 and 70 nm and they are mixed with nanoparticles. The effect of pulse to pulse separation rate on the size of the magnetic fibrous structure and the magnetic strength was reported. X-ray diffraction (XRD) analysis revealed metallic and oxide phases in the nanostructure. The growth of magnetic nanostructure is highly recommended for the applications of magnetic devices like biosensors and the results suggest that the pulsed-laser method is a promising technique for growing nanocrystalline magnetic nanofibers and nanoparticles for biomedical applications.

## Introduction

Nanomaterials field is of current interest because it studies materials with morphological features on the nanoscale. Nanosized materials show distinctive properties compared with bulk materials [1-3]. In particular, magnetic nanostructures have recently attracted much attention because of their intriguing properties that are not displayed by their bulk or particle counterparts. These nanostructures are potentially useful as active components for ultrahigh-density data storage, as well as in the fabrication of sensors and spintronic devices [4].

The growth of nanofibers using ultrafast laser offers advantages of high resolution, high throughput, uniformity, localized heating, simplicity, and reproducibility [5-8]. The time scale of materials heating and cooling of traditional thermal processes is significantly higher than that with femtosecond laser irradiation [9]. The rapid absorption of energy leads to efficient material removal before significant heat diffusion to the substrate occurs. Femtosecond laser radiation has already been used to fabricate nano-sized spikes of semiconductor [10], metallic [11,12], and dielectric surfaces [13] in vacuum.

Magnetic neodymium-iron-boron (NdFeB) nanofibers and nanoparticles have become one of the hotspots in the research field of magnetic materials to meet the demand for miniaturization of electronic components in recent years, and have been successfully prepared by various routes like the sol-gel auto-combustion method [14], co-precipitation [15], hydrothermal method [16], reverse micelles [17], microemulsion method [18], alternate sputtering [19], pulsed-laser deposition [20], and so on. However, until now there have been no reports on the synthesis and magnetic properties of NdFeB ferrite nanofibers in literatures.

In the present study a magnetic weblike fibrous nanostructure is formed due to the agglomeration of the bulk quantity of nanoparticles created during laser ablation at mega hertz pulse frequency. A distinct characteristic of the fibrous nanostructures is that particles are fused and the agglomeration shows certain degree of organization, unlike the random stacking of particles observed at femtosecond laser ablation at pulse frequency in kilohertz and hertz regime. The effect of pulse repetition rate on the nanofibers size and hence the magnetization was also investigated. The nanostructures were characterized by scanning electron microscopy (SEM), transmission electron microscopy (TEM), energy-dispersive X-ray (EDX), X-ray diffraction (XRD), and magnetic force microscopy (MFM). The mechanism of formation is explained by the well-established theory of vapor condensation induced by ultrafast laser ablation. Also, the fibrous nanostructures have relatively uniform diameters (30-90 nm) and did not observe a wide range of variation in size distribution. This agrees with the characteristics of nanoparticle formation through homogenous nucleation, which tends to generate monosized nanoparticles.

## Experimental details

The laser source is a diode-pumped Yb-doped fiber oscillator/amplifier system (Clark MXR Inc.) capable of producing an average power of 15.5 W with pulse repetition frequency between 200 kHz and 25 MHz. A neodymium-iron-boron magnetic specimen of 1" ± 0.008" length by 1" ± 0.008"; width by 0.1" ± 0.005" thickness was cut into four pieces of same size. The tetragonal Nd_2_Fe_14_B crystal structure has exceptionally high uniaxial magnetocrystalline anisotropy. This gives the compound the potential to have high coercivity. To generate magnetic nanofibers, the first piece of the magnetic specimen was irradiated with laser using 1040 nm wavelength with 15 W power and a pulse repetition rate of 4 MHz. The experiment was repeated to generate nanofibers on the specimen using the same power and wavelength with frequencies of 8, 13, and 26 MHz. The irradiated sample was characterized using SEM, TEM, EDX, and XRD analysis.

## Results and discussion

**Figure 1:**
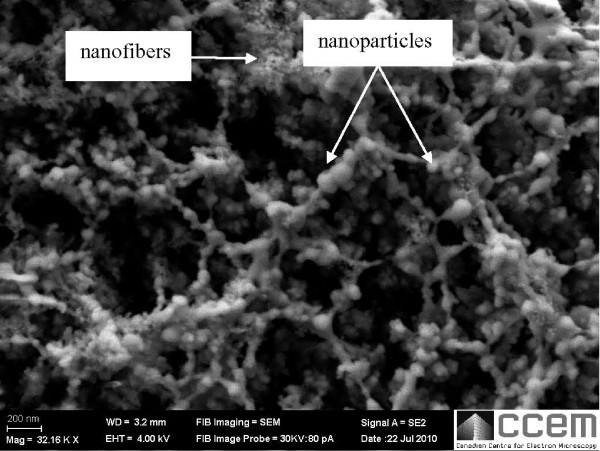
**SEM image of magnetic nanofibrous structure and nanoparticles on NdFeB substrate irradiated with femtosecond laser at 26 MHz repetition rate and 15 W average power**.

The energy of the femtosecond laser is delivered into the material in a short time scale that absorption occurs at nearly solid-state. The energy is first deposited in the electronic subsystem within a layer of thickness of tens of nanometer. Enough energy is absorbed to produce macroscopic ablation when the density of the free electrons exceeds a certain threshold [21]. The ionized material is removed away from the surface in the form of expanding high pressure plasma. The plasma remains confined close to the specimen surface at atmospheric pressure. Condensation of vapor in the plume leads to the generation of nanoparticles. Some of these nanoparticles aggregate and then get deposited on the surface of the specimen [8]. Vapor condensation starts with nucleation, proceeds with growth of supercritical nucleus and come to a halt due to quenching. For nanoparticles to aggregate and form fibrous structure, a continuous supply of vapor is required to the expanding plume to maintain the nucleus density. Hence nanoparticles generated from the successive laser pulse are fused to the particles created from the previous laser pulse that are still above the melting temperature and grow as nanofibrous like structure as shown in Figure 1. Dipole-dipole interactions then trigger anisotropic chain growth under the influence of serendipitous Brownian collisions, attractive van der Waals, as well as the residual electrostatic repulsions that maintain colloidal stability [22]. The energy barrier to surface reorganization is overcome over the very high temperature, resulting in the rapid onset of self-assembly of the nanoparticle chains (or nanofibers).

**Figure 2:**
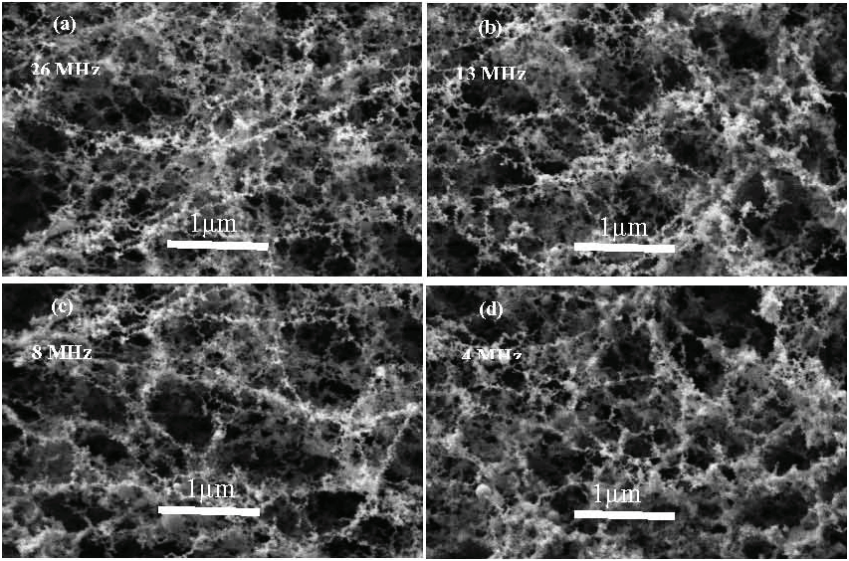
**SEM images of the generated nanofibers (a) 26 MHz, (b) 13 MHz, (c) 8 MHz, and (d) 4 MHz**.

**Figure 3:**
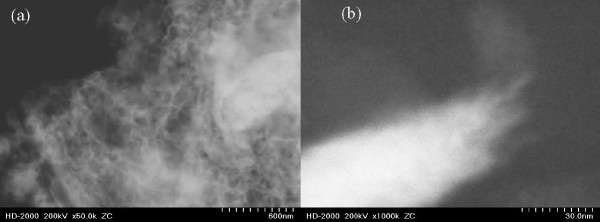
**TEM images of magnetic nanofibers generated by femtosecond laser at 26 MHz pulse repetition rate and 15 W power**.

**Figure 4:**
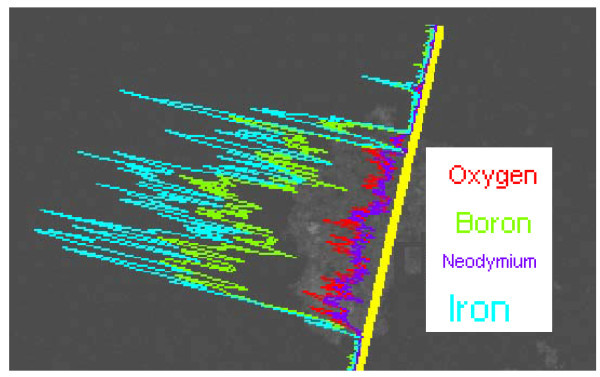
**EDX analysis of magnetic nanofibers structures**.

The laser pulse repetition rate plays a critical role in the formation of nanofibrous like structure. Figure 2 shows SEM images of the magnetic weblike nanofibers generated at 4, 8, 13, and 26 MHz pulse repetition rate. The average diameters of the generated nanofibers were around 70, 60, 45, and 30 nm, respectively. Figure 3a shows the TEM image of magnetic nanofibers at 26 MHz pulse repetition rate and Figure 3b shows a single magnetic nanofiber generated by femtosecond laser. It depicts that magnetic nanofibers possess weblike structures with diameter not more than 30 nm. Further EDX analysis of the irradiated surface shows existence of oxygen as shown in Figure 4 which indicates, besides the percentage of oxygen to neodymium-iron-boron, the existence of oxidized magnetic nanofibers [23].

**Figure 5:**
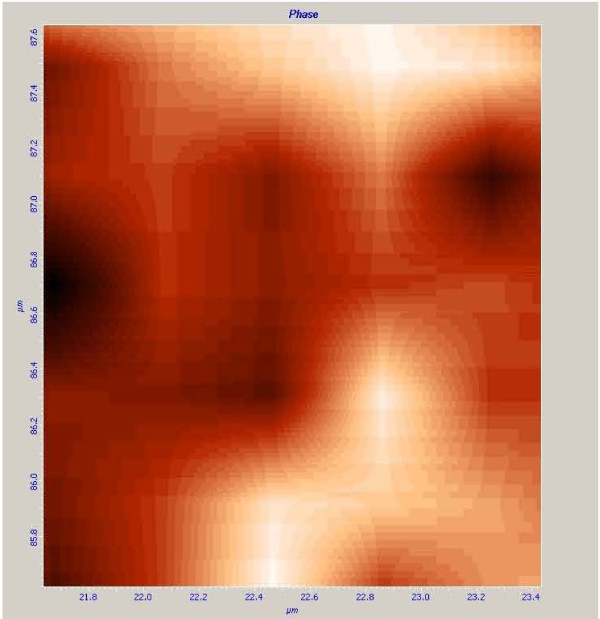
**MFM image of NdFeB nanofibrous structures formed upon irradiation of laser at 26 MHz pulse repetition rate**.

During ablation, the ionized material is removed away from the surface in the form of expanding high pressure plasma. The temperature of the plasma is above the melting temperature and hence Curie temperature of the magnet. Thus the irradiated spot and the surrounding area where temperatures above 400°C will be demagnetized while the rest of the sample remains magnet. The plasma remains confined close to the specimen surface at atmospheric pressure. Condensation of vapor in the plume leads to the generation of nanoparticles which move in the direction where the paramagnetization potential energy is minimized [24]. The nanoparticles travelled perpendicular to the direction of the magnetic field and then aggregate and get deposited on the surface of the specimen [25]. The generated nanofibers remagnetized when its temperature reduced below the Curie temperature of the sample to form magnetic nanofibers. The total magnetization of a nanofiber is given by the vectorial sum of all single magnetic moments of the atoms [24]. As for the atomic magnetic moments in generated nanofibers, the average magnetization will be zero in the absence of magnetic field since all magnetic moments are randomly directed in space. When a magnetic field is applied by the substrate, the magnetic moments will orient in the direction of the field and give rise to a net magnetization of the nanofibers. Magnetic field microscopy, from NT-MDT, (MFM) image of the weblike nanofibers structures generated at 26 MHz is shown in Figure 5. The NdFeB nanofibers exhibit magnetic properties (darker parts) as shown in the MFM image of Figure 5 which are distinguishable from the background (brighter parts).

The laser pulse repetition rate plays a critical role in the formation of magnetic nanofibrous structure [26]. In order for nanoparticles to aggregate and form fibrous structure, a continuous supply of vapor is required to maintain the nucleus density of the expanding plume. Nanoparticles generated from the successive laser pulse are fused to the particles created from the previous laser pulse that are still above the melting temperature and grow as nanofibrous like structure as shown in Figure 1. As the pulse repetition rate of the femtosecond laser increases, the time between successive pulses decreases which gives less time for clusters to agglomerate and generate nanofibers with smaller diameter. It is evident from the SEM images shown in Figure 2a-d that smaller size nanofibers was generated with the increase of the laser pulse repetition rate.

**Figure 6:**
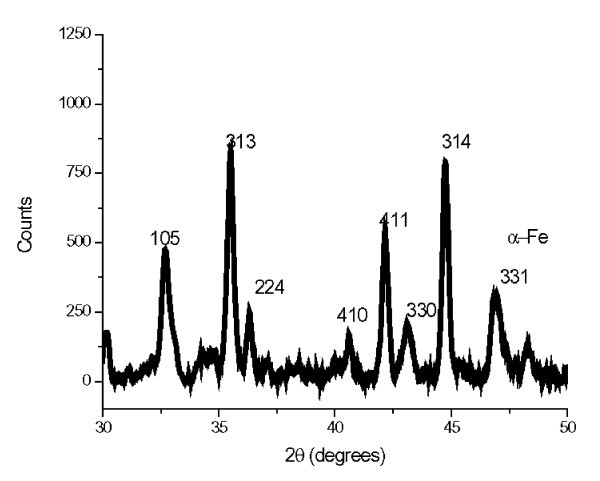
**XRD pattern of NdFeB magnetic nanofibers generated at 26 MHz and 15 W**.

**Figure 7:**
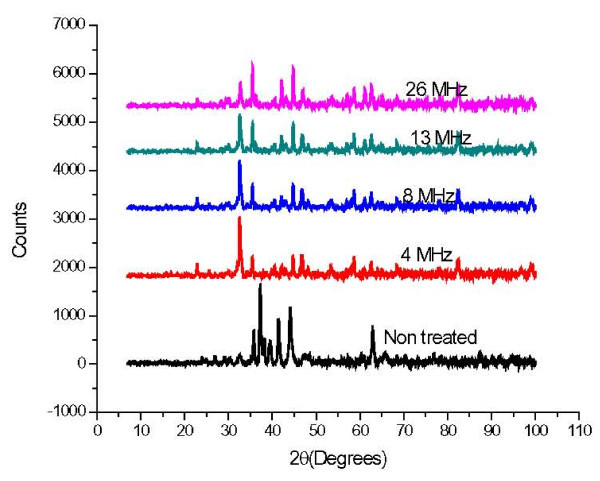
**XRD patterns for NdFeB magnetic nanofibers generated at 4, 8, 13, and 26 MHz**.

Characterization was performed using XRD as a function of femtosecond laser pulse repetition rate. Figure 6 shows XRD pattern of NdFeB magnetic nanofibers generated by femtosecond laser at 26 MHz and a power of 15 W. The average nanofibers size is about 28.5 nm estimated from the XRD peaks using the Scherrer formula [25]. This value is consistent with nanofiber size obtained by TEM analysis as shown in Figure 3. In comparison, the size of nanofibers prepared using the conventional methods is around 40 nm which is slightly bigger than our method and do not have the weblike structure [27]. Figure 7 shows the XRD patterns for magnetic nanofibers generated at 4, 8, 13, and 26 MHz, respectively. For the non-irradiated area in Figure 7, no diffraction peaks indexed by the Nd2Fe14B phase were observed. However, the peaks from Nd2Fe14B phase can be observed clearly in the samples irradiated with femtosecond laser. For the area irradiated with laser at 26 MHz, the peak from α-Fe was mainly found. Therefore, it is considered that the α-Fe peak is attributed to the surface oxidation and it is existed on the surface of the sample.

**Figure 8:**
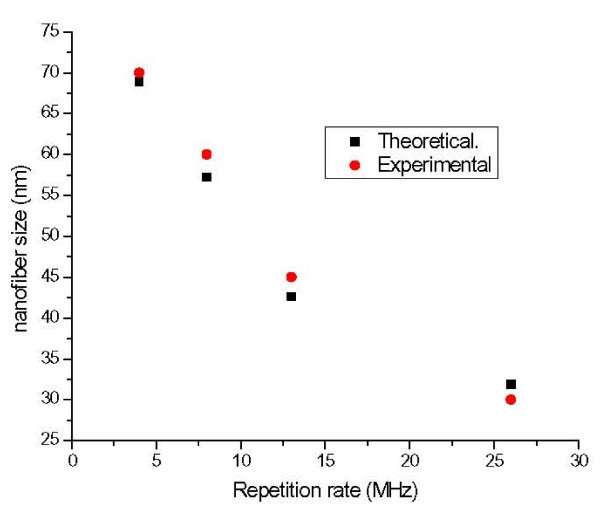
**Theoretical and experimental magnetic nanofibers size as a function of femtosecond laser pulse repetition rate**.

Figure 8 shows the experimental and theoretical relationship between laser pulse repetition rate and magnetic nanofibers size. The nanostructures were generated as a result of nanoparticle agglomeration. As the laser pulse repetition rate increases, the pulse to pulse duration will be shorter and hence less time for agglomeration process is available which results in smaller size fibrous nanostructure [23]. The average nanofiber size can be estimated from the Sherrer equation [28]:(1)

where *r *is the nanofiber size, λ is the X-ray wavelength, *B *is the full width at half maximum of the peak (FWHM), and θ is the diffraction angle. From the diffraction peaks in Figure 7, the average nanofiber size was estimated using the above equation and plotted in Figure 8. Those calculations are close to our experimental results as shown in the figure.

The metastable Nd-rich phase is a grain-boundary phase which has an FCC structure. This grain boundary phase exhibits a characteristic contrast which is similar to a metastable high-pressure phase observed previously as FCC γNd [29]. The structure of the phase is, however, closely related to that of NdO and it was frequently reported that oxygen content is fundamental in the formation of this phase [30]. However, oxygen-containing FCC phases as shown in Figure 4 were observed only at high temperatures. Therefore, oxygen presence is not critical for the formation of the FCC phase, although at higher temperature this phase may absorb oxygen more easily than other phases because of the high Nd content. Moreover, oxygen can probably stabilize this metastable phase and at higher temperature it can transform into the stable NdO oxide. It was noticed, however, more than three phases can coexist at a given temperature (e.g., at melting point) only if the fourth element was introduced into the ternary system, i.e., oxygen in Nd-Fe-B system [31]. The FCC phase is presumably a metastable phase with a structure close to the short-range order in the Nd-rich amorphous phase [32]. It probably forms from the undercooled substrate with lower melting point than Nd_2_Fe_14_B or from the amorphous phase produced at grain boundaries during the laser ablation process.

**Figure 9:**
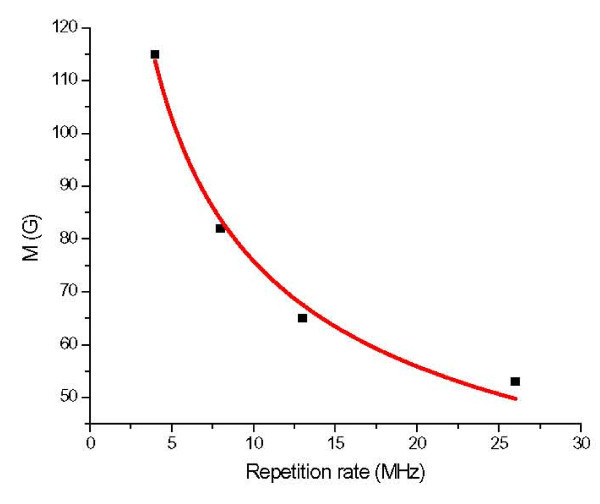
**Magnetic strength *M *as a function of laser pulse repetition rate**.

Figure 9 shows the typical variations of magnetic strength *M *as a function of laser repetition rate for the NdFeB nanofibers grown at room temperature. The thickness of the generated fibers layer in all of the four pieces were the same [23], however, the morphology of the nanostructures would be changed because of the change in nanofibers size caused by the change in repetition rate. The data were for the samples measured with a Guassmeter along the in-plane direction. The figure indicates that at higher repetition rates, the *M *of the nanofibrous structure get lower due to the presence of an abundant amorphous phase which also shows lower coercivity. The relatively large coercivities of nanofibrous structures were due primarily to their specific morphology. Theory has predicted that a system containing magnetic dipoles that are arranged into a linear chain will exhibit an increase in coercivity [33]. Our results seemed to be consistent with this prediction as long as dipole-dipole interactions between grains played the dominant role in the magnetization process. The NdFeB grains contained in each nanofiber were actually aligned along its long axis, and the dipole-dipole interactions between grains tended to line up all magnetic dipoles along the same axis.

## Conclusions

We introduced synthesis of NdFeB magnetic fibrous nanostructure and nanoparticle on bulk substrate using femtosecond laser radiation under ambient conditions. The phase structures and microstructures have been investigated using XRD, SEM and EDX analysis. The magnetic nanofibers were grown in the order of few nanometers and organized themselves in weblike structures. Magnetic nanoparticles with diameter in the order of few nanometers were attached to the nanofibrous structure. Increasing the repetition rate of the femtosecond laser results in increasing the number of pulses and hence decreases size of the generated magnetic nanofibers. Increasing repetition rate of the femtosecond laser results in generating smaller size magnetic nanofibers. The magnetic strength of the generated nanofibers can be controlled by changing the repetition rate of the femtosecond laser. These magnetic nanofibers may be utilized in many applications, such as magnetic devices, carriers, tissue engineering materials, and drug delivery.

## Abbreviations

EDX: energy-dispersive X-ray; MFM: magnetic force microscopy; NdFeB: neodymium-iron-boron; SEM: scanning electron microscopy; TEM: transmission electron microscopy; XRD: X-ray diffraction.

## Competing interests

The authors declare that they have no competing interests.

## Authors' contributions

MA carried out laser processing of the samples, characterisation and drafted the manuscript. KV conceived of the study, and participated in its design and coordination. BT conceived of the study, and participated in its design and coordination. All authors read and approved the final manuscript.
